# A Rare Case of Congenital Generalized Lipodystrophy

**DOI:** 10.7759/cureus.64276

**Published:** 2024-07-10

**Authors:** Shiji Chalipat, Om Prasanth Reddy Avuthu, P. Sindhura, Shailaja V Mane

**Affiliations:** 1 Pediatric Neurology, Dr. D. Y. Patil Medical College, Hospital and Research Centre, Pune, IND; 2 Pediatrics, Dr. D. Y. Patil Medical College, Hospital and Research Centre, Pune, IND

**Keywords:** subcutaneous adipose tissue, rare autosomal recessive disorder, genetic screening, pediatrics neurology, pediatrics & neonatology, pediatric genetics

## Abstract

Congenital generalized lipodystrophy type 2 (CGL2) is a rare autosomal recessive disorder characterized by the near-total absence of adipose tissue, leading to various metabolic complications. We present the case of a one-year-old male who exhibited progressive abdominal distension from six months of age. Physical examination revealed distinctive features including triangular facies, hypertelorism, an emaciated appearance with absent buccal fat, and hepatosplenomegaly. Laboratory investigations showed elevated transaminases and a deranged lipid profile, while imaging confirmed hepatosplenomegaly without systemic anomalies. A liver biopsy indicated macrovesicular steatosis and impending cirrhosis. Genetic testing revealed a homozygous pathogenic variant in the BSCL2 gene (c.604C>T), confirming CGL2. The child is under regular follow-up, with genetic counseling provided to the parents. This case underscores the importance of early recognition, genetic diagnosis, and regular monitoring in managing this rare condition.

## Introduction

Berardinelli-Seip congenital lipodystrophy (BSCL), also known as congenital generalized lipodystrophy (CGL), is a rare genetic disorder characterized by the near-complete absence of adipose tissue from birth [1,2]. It was first described in 1954 by Berardinelli and later again in 1959 by Seip [3,4]. This condition manifests with a distinctive clinical phenotype, including marked muscularity, prominent veins due to lack of subcutaneous fat, and characteristic facial features such as triangular facies and hypertelorism [1,5]. BSCL/CGL is inherited in an autosomal recessive pattern and is typically diagnosed early in life due to its striking physical appearance and associated metabolic disturbances [1].

CGL, a rare autosomal recessive disorder, affects individuals across all ethnicities, with a global prevalence estimated at approximately one in every 10 million people [6]. Families with CGL are categorized into types I, II, III, and IV, each associated with distinct genetic mutations [7]. Type 1 is linked to mutations in the AGPAT2 gene on chromosome 9q34, while type 2 is associated with mutations in the BSCL2 gene on chromosome 11q13. The hallmark of CGL is the near absence of adipose tissue, leading to abnormal storage of fats in metabolically active tissues such as muscle and liver. This aberrant fat distribution results in severe insulin resistance, often progressing to diabetes mellitus. Metabolic consequences include hypertriglyceridemia predisposing to hepatic steatosis, which can progress to cirrhosis and liver failure, a significant cause of mortality. Additionally, individuals with CGL frequently develop hypertrophic cardiomyopathy, contributing to cardiac failure as a cause of death [7].

Clinical manifestations of CGL encompass the loss of subcutaneous fat, pronounced muscular hypertrophy, elongated extremities (feet, hands, and jaw), an acromegalic appearance, and acanthosis nigricans [8]. Patients may also present with accelerated growth, hepatosplenomegaly, and evidence of advanced bone age. Laboratory findings typically reveal signs of liver dysfunction such as elevated aminotransferases, dyslipidemia characterized by high triglycerides and low high-density lipoprotein (HDL) cholesterol, and possible elevations in total cholesterol and low-density lipoprotein (LDL) cholesterol levels [8,9]. The diagnosis of CGL relies on clinical phenotype assessment complemented by genetic testing to confirm the specific variant, guiding appropriate management and genetic counseling for affected families [8].

## Case presentation

We report the case of a one-year-old male child, the only child born to parents in a third-degree consanguineous marriage. The child presented with progressive abdominal distension noted since six months of age, without significant symptoms suggestive of systemic involvement. The perinatal period was uneventful, though the child exhibited mild motor developmental delay and had no significant family history.

**Figure 1:**
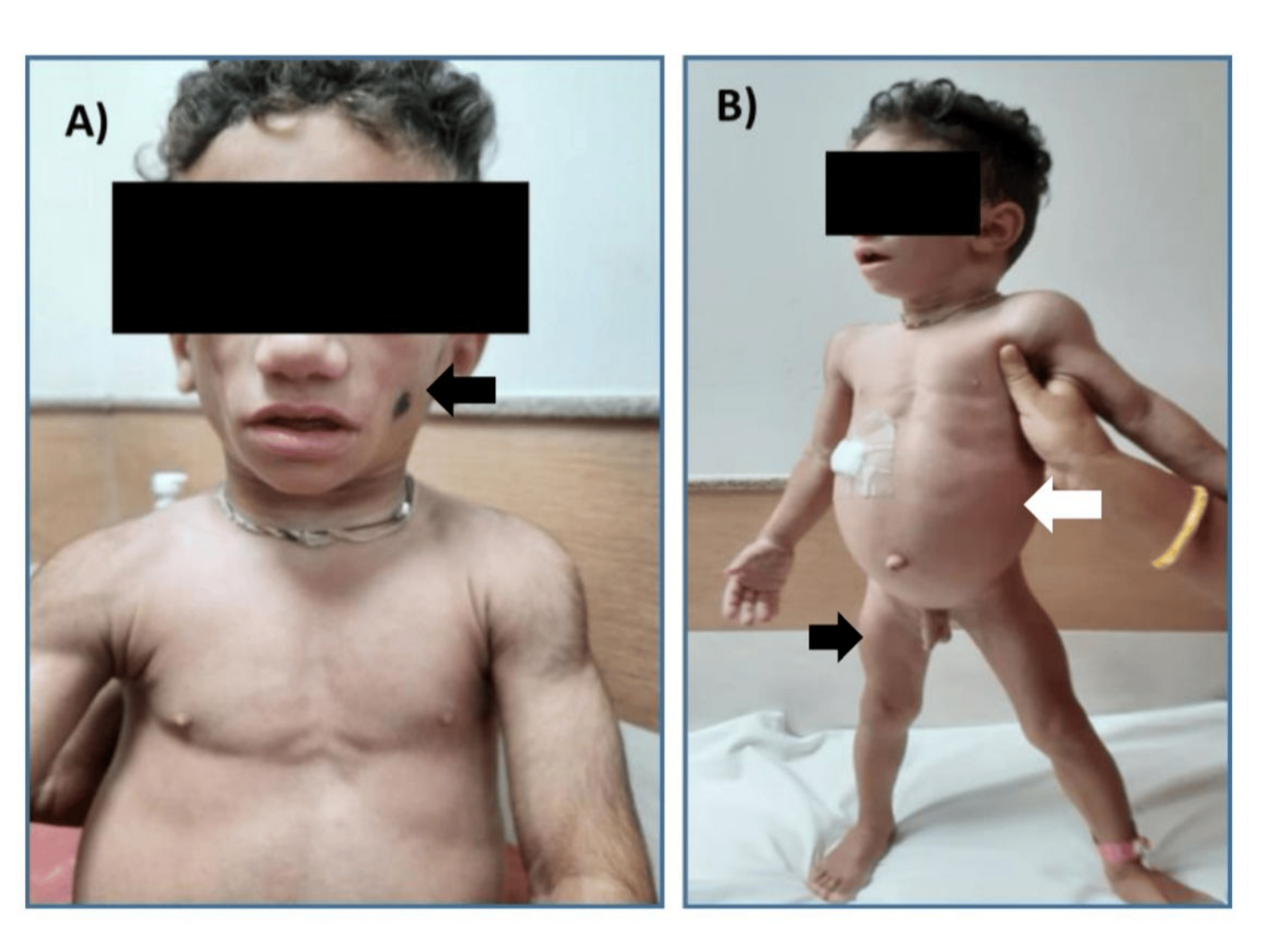
Clinical picture of the patient. A) Loss of subcutaneous fat and loss of buccal pad of fat (indicated by black arrow). B) Protuberant abdomen (indicated by white arrow) and prominent musculature (indicated by black arrow).

The child, aged one year, weighed 10 kg (>75th percentile), measured 78 cm in height (75th-90th percentile), and had a head circumference of 47 cm (median to +1 SD). Physical examination revealed triangular facies, an emaciated appearance with an absent buccal pad of fat, hypertelorism, down-slanting eyes, and posteriorly placed ears. The child also had thick, curly scalp hair, excessive body hair, prominent subcutaneous veins, non-pinchable skin, and absent subcutaneous fat. His muscular body exhibited brachydactyly (Figure 1).

The abdomen was distended with an everted umbilicus and normal external genitals. Additionally, there was firm, non-tender hepatomegaly with a liver span of 13 cm and grade 2 splenomegaly without free fluid. Other systemic examinations and an ophthalmologic evaluation were normal. Considering the clinical presentation, differential diagnoses included storage disorders, Hutchinson-Gilford progeria syndrome, and acquired CGL. Laboratory investigations showed a normal hemogram and elevated transaminases (SGOT: 117 U/L and SGPT: 220 U/L). The lipid profile revealed a total cholesterol level of 184 mg/dL, serum triglycerides of 126 mg/dL, LDL cholesterol of 139 mg/dL, and HDL cholesterol of 20 mg/dL. Fasting blood sugar was normal at 77 mg/dL, as was serum uric acid at 5.3 mg/dL and CPK-NAC at 83. Liver function tests indicated a total bilirubin level of 0.26 mg/dL and an alkaline phosphatase level of 268 U/L (Table 1).

**Table 1:** Laboratory investigations with their respective normal reference ranges and observed values. CRP, C-reactive protein; LDL, low-density lipoprotein; HDL, high-density lipoprotein

Investigation	Normal values	Observed values
Hb (g/dL)	11-14.5	11.3
Total leukocytes (mm^3^)	4000-12000	20900
Platelets (/µL)	150000-410000	422000
CRP (mg/L)	<3	2.3
Total bilirubin (mg/dL)	<1	0.26
SGOT (U/L)	10-40	117
SGPT (U/L)	5-45	220
ALP (U/L)	140-560	268
Fasting blood sugar	60 mg/dL-100 mg/dL	77 mg/dL
Serum uric acid	2.2 mg/dL-5.5 mg/dL	5.3 mg/dL
Total cholesterol	<170 mg/dL	184 mg/dL
Serum triglycerides	<100 mg/dL	126 mg/dL
LDL cholesterol	<110 mg/dL	139 mg/dL
HDL cholesterol	>45 mg/dL	20 mg/dL
HbA1C	<8.5%	5.5
CPK-NAC	<90 U/L	83 U/L

**Figure 2:**
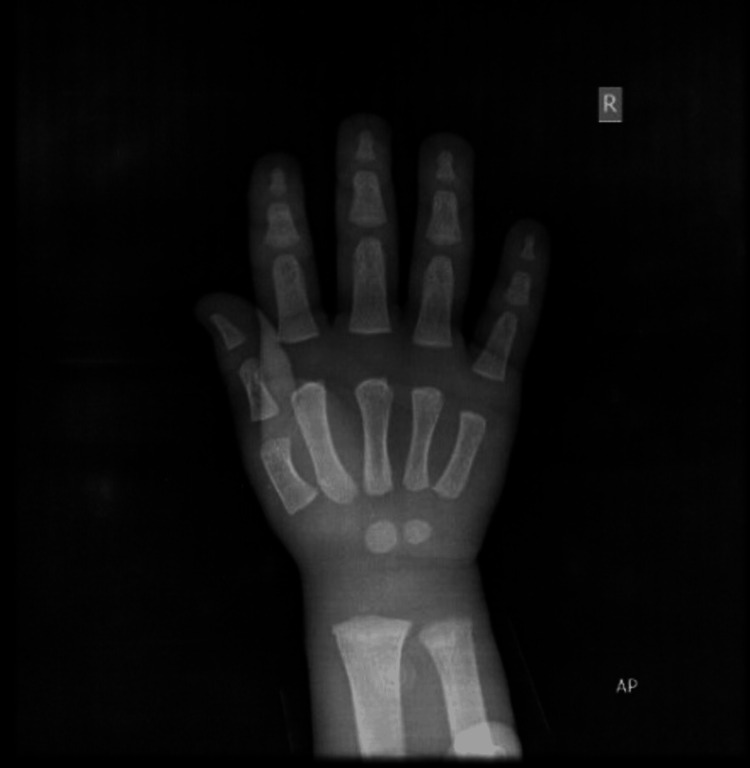
X-ray of the right wrist and hand, which showed a bone age corresponding to the chronological age of one year.

Imaging and other studies included an X-ray of the right wrist and hand, which showed a bone age corresponding to the chronological age of one year (Figure 2).

An abdominal ultrasound revealed hepatosplenomegaly, and echocardiography results were normal. A liver biopsy indicated rarified hepatocytes with focal macrovesicular steatosis and impending cirrhosis. Clinical exome sequencing identified a homozygous pathogenic variant on the BSCL2 gene (exon 5, variant c.604C>T), confirming the diagnosis of CGL2.

The child is under regular follow-up with monitoring of liver function, lipid profile, and growth parameters. Genetic counseling has been provided to the parents to discuss the hereditary aspects of the condition and its implications.

## Discussion

Attributed to mutations in the BSCL2 gene encoding Seipin, a protein crucial for adipocyte differentiation and lipid metabolism regulation, congenital lipodystrophy type 2 (BSCL2) represents one of the most severe forms [1]. The absence of adipose tissue in BSCL/CGL results in severe metabolic complications, primarily due to insulin resistance and dyslipidemia. Individuals with this condition often develop early-onset diabetes mellitus, hypertriglyceridemia, and hepatomegaly [10]. Unlike AGPAT2 mutations, which primarily affect adipose tissue, BSCL2 mutations can impact various tissues, potentially contributing to intellectual disabilities due to Seipin's expression in the brain. In our presented case, the child exhibited a mild motor developmental delay without an intellectual disability. A report by Agarwal et al., 2003, has shown that mutations in the BSCL2 gene may cause an earlier onset of diabetes and a higher incidence of mild mental retardation [11]. Another interesting report by Windpassinger et al., 2004, shows that mutations in the BSCL2 involving N88S and S90L specifically impair the glycosylation of Seipin, leading to the formation of protein aggregates and subsequent neurodegeneration. These findings underscore the critical role of Seipin in ER function and highlight how mutations in BSCL2 can disrupt cellular processes, contributing to the pathogenesis of neurological complications in individuals affected by this rare genetic disorder [12].

Management of BSCL/CGL involves a multidisciplinary approach, focusing on metabolic control through dietary management, pharmacological therapies to manage insulin resistance and dyslipidemia, and monitoring for potential complications such as liver steatosis and cardiovascular disease. Genetic counseling is essential for affected individuals and their families to understand the inheritance pattern and the risks associated [13].

The clinical diagnosis of CGL is based on major criteria such as lipoatrophy with muscular hypertrophy, acromegaloid features, hepatomegaly, and signs of insulin resistance like acanthosis nigricans and elevated triglyceride levels [7]. Our case displayed these major criteria while lacking minor criteria such as hypertrophic cardiomyopathy, psychomotor retardation, hirsutism, precocious puberty in females, and bone cysts. This comprehensive clinical and genetic understanding underscores the complex manifestations and diagnostic challenges associated with CGL.

## Conclusions

This case highlights the clinical features, laboratory findings, and genetic diagnosis of CGL2. Early recognition and genetic confirmation are crucial for managing complications and providing appropriate genetic counseling. Management includes a multidisciplinary approach with diet modifications and pharmacotherapy; there is no cure for this condition, and management mostly remains as supportive care. Regular follow-up is essential for monitoring and managing the metabolic and hepatic complications associated with this condition.
